# Improving seed germination: effect of stratification and dormancy-release priming in *Lonicera insularis* Nakai

**DOI:** 10.3389/fpls.2024.1484114

**Published:** 2024-11-06

**Authors:** Jihun Lee, Kyungtae Park, Hamin Lee, Bo-Kook Jang, Ju-Sung Cho

**Affiliations:** ^1^ Division of Animal, Horticultural and Food Sciences, Chungbuk National University, Cheongju, Republic of Korea; ^2^ Department of Demilitarized Zone (DMZ) Forest Biological Resources Conservation, Korea National Arboretum, Yanggu, Republic of Korea; ^3^ Department of Horticulture, Sunchon National University, Suncheon, Republic of Korea

**Keywords:** embryo growth, endemic plant, germination temperature, morphological dormancy, seed propagation

## Abstract

*Lonicera insularis* Nakai is endemic plant predominantly distributed along the coastlines of Ulleung-do and Dok-do in South Korea, and is significant for medicinal and ornamental uses. However, previous studies reported that *L. insularis* seeds exhibit morphological dormancy (MD), causing them to germinate only at specific temperatures. Therefore, this study aimed to classify the dormancy type of *L. insularis* seeds, expand the germination temperature range, and confirm the industrial applicability through dormancy-release (DR) priming. The seeds of *L. insularis* exhibited no inhibition of water absorption by the seed coat, with 85.0% germination observed at 15°C, similar to the viability (84.0%). Initially, the initial seeds of *L. insularis* exhibited an embryo-to-seed (E:S) ratio of 0.24 during dispersal, which increased to 0.81 by day 18 at 15°C, indicating that the seeds exhibit MD with underdeveloped embryos. Treatments with GA_3_ and GA_4 + 7_ to overcome MD and expand the germination temperature range did not significantly enhance germination. However, seeds that grew embryos through stratification for 2 weeks at 15°C effectively expanded the germination temperature range. Additionally, DR-primed seeds maintained the expanded germination temperature range even after redrying, confirming the commercial potential of DR-primed seeds. Therefore, this study represents the first application of a novel approach to alleviate dormancy in *L. insularis* seeds through the integration of stratification and priming techniques, termed dormancy-release priming. This approach resulted in a significant increase in germination and expanded the effective temperature range for seed germination. These findings suggest that dormancy-release priming could be a valuable method for producing non-dormant seeds, enhancing the potential for *L. insularis* distribution and industrial applications.

## 1 Introduction

The genus *Lonicera* consists of over 200 species of arching shrubs or small trees that are primarily cultivated as ornamental crops (Theis et al., 2008). *L. insularis*, which belongs to the Caprifoliaceae family, is primarily distributed along the coastlines of Ulleung-do and Dok-do in South Korea, flowering from May to June (Yu et al., 2022). Native to specific regions of East Asia, *L. insularis* is a valuable resource for ornamental and medicinal purposes, and its extracts have been used as Chinese herbal remedies for treating inflammation since ancient times (Ryuk et al., 2012; Yuan et al., 2012; Kim et al., 2016). *L. morrowii*, a species genetically similar to *L. insularis*, has been suggested as the progenitor of *L. insularis* through morphological and geographical studies (Jeong et al., 2014). The genetically similar *L. morrowii* was introduced to the United States as an ornamental plant around 1875 but has since become invasive, negatively impacting ecosystems by affecting nesting songbirds, herpetofauna, spider richness, and invertebrate biomass (Love and Anderson, 2009). These plant invasions pose serious challenges to the environment, ecosystem, and economy worldwide (Chen et al., 2009). In contrast, *L. insularis* is endemic plant in South Korea and limited to the island of Ulleung-do (Jeong et al., 2014; Jung et al., 2014). Numerous endemic species, including *L. insularis*, have experienced unprecedented rates of decline owing to human effects on natural ecosystems, climate change, and ecological collapse (Hurdu et al., 2022). Preserving plant diversity, specifically in island ecosystems, is crucial. Therefore, research on the propagation of various native plants is necessary to address conservation challenges. Seed propagation plants through seeds is a cost-effective and practical method for conserving threatened plant species (Yucel, 2022). Additionally, understanding seed germination and duration of existing plant seeds is directly related to the successful propagation techniques. However, unlike widely propagated crop plants, numerous wild plant species face challenges in propagation owing to dormancy, making research on germination an essential first step in their growth (Kildisheva et al., 2020). Therefore, it is necessary to assess the germination characteristics of *L. insularis* seeds for efficient propagate this valuable resource plant.

The majority of wild plant species have evolved various dormancy mechanisms to prevent germination under unfavorable environmental conditions for plant growth (Zhang et al., 2021). A seed is the structure in which a usually fully developed plant embryo is dispersed, and which enables the embryo to survive the period between seed maturation and seedling establishment, thereby ensuring the initiation of the next generation (Koornneef et al., 2002). While seed dormancy is biologically useful in natural environments, it poses challenges for the use of crops or ornamental plants, often requiring weeks to over a year to break dormancy (Heather et al., 2010). Seed dormancy and germination are controlled by various environmental factors, such as light, temperature, and the duration of seed storage (after ripening) (Bewley, 1997). Dormancy types are classified into five categories: physiological dormancy (PD), morphological dormancy (MD), morphophysiological dormancy (MPD), physical dormancy (PY), and combinational dormancy (PY+PD) (Baskin and Baskin, 2014).

Previous studies have classified *L. insularis* seeds as having MD, with embryo growth occurring at 15°C (Park et al., 2019). MD facilitates germination within 4 weeks but involves an underdeveloped embryo (Lee et al., 2015). *Lonicera* species are mostly classified as those exhibiting MD and MPD, which are characterized by underdeveloped embryos (Hidayati et al., 2000; Phartyal et al., 2009; Santiago et al., 2013; Maola et al., 2019). Seeds with MD can reduce the dormancy-breaking period through gibberellic acid (GA) treatment and stratification (Luo et al., 2023). These seeds with MD have underdeveloped embryos that require time to grow to a critical length [embryo-to-seed (E:S) ratio ≥ 0.8] before germination, and can only germinate at specific temperatures (Baskin and Baskin, 2004). And these embryos are not physiologically dormant; they simply require time to grow and germinate (Finch-Savage and Leubner-Metzger, 2006). Germination timing depends on the interaction between the dormancy state and environmental response. Once seed dormancy is alleviated, the range of environmental conditions that facilitate germination expands (Murphey et al., 2015). Additionally, the germination temperature range significantly affects the species ecology, and a broader germination temperature range facilitates the production of more seedlings (Baskin et al., 2022). This significantly facilitates the utilization of seeds from valuable resource plants with extended germination temperature ranges, by alleviating seed dormancy. Therefore, further research is required to alleviate the dormancy of seeds with MD and expand their germination temperature range.

Seed dormancy negatively affects agriculture, and the degree of dormancy directly affects crop yield and quality, necessitating research on seed technologies to utilize the species effectively (Fu et al., 2024). Priming technology, which involves activating seed germination metabolism and subsequent redrying, is used by numerous agricultural seed companies to enhance germination capacity, resistance to biotic and abiotic stresses, and crop yield (Paparella et al., 2015). Additionally, seed priming helps to regulate various types of seed dormancy and enhances seed vigor (Rao et al., 2019). However, despite its extensive use in the agricultural industry and significant benefits, there are few applications of seed priming for native plants in areas, such as species utilization and ecological restoration (Pedrini et al., 2020). Therefore, to apply priming to dormant seeds, it is necessary to develop novel techniques that combine dormancy-release (DR) treatment with priming that involves redrying after water absorption, which may be useful for species utilization and ecological restoration. These novel priming techniques can produce seeds that are better adapted to various conditions, thereby offering solutions for ecological restoration and the agricultural industry in response to climate crises (Singh et al., 2020). Specifically, expanding the application of these technologies to native species can offer significant ecological benefits, supporting species preservation and the restoration of degraded habitats.

Therefore, this study aimed to classify the dormancy types of *L. insularis* seeds, alleviate dormancy to expand the germination temperature range, and confirm the potential of DR priming to enhance germination.

## 2 Materials and methods

### 2.1 Plant material

*L. insularis* seeds were collected from habitats in Ulleung-gun, Gyeongsangbuk-do, South Korea in September 2021. At the time of collection, the temperature in the region ranged from 15.8 – 29.1°C, and only seeds that were sufficiently mature were harvested. These seeds were provided from the National Institute of Biological Resources and only those that were sufficiently mature at the time of collection were harvested. The seeds collected immediately after harvest were dried in the shade, subsequently selected, and sealed in a plastic bag with silica gel, then stored at stored in the dark at 4°C until used in the experiment.

### 2.2 Seed characteristics

The basic characteristics of the seeds, including 100-seed weight, length and width, E:S ratio, and viability, were assessed. The 100-seed weight was determined by weighing 100 seeds in four replicates using a microbalance (AS200; Radwag, Radom, Poland). The length and width of 10 seeds were measured using a stereomicroscope (SZ61; OLYMPUS, Tokyo, Japan) equipped with a complementary metal-oxide-semiconductor (CMOS) camera (eXcope F-630; Dixi Sci., Daejeon, Korea). To assess the presence of MD, 10 seeds were cross-sectioned using a double-edged razor, and the E:S ratio was measured using a stereomicroscope, with the average value recorded. Seed viability was assessed by soaking 50 seeds in distilled water at 20°C in the dark for 24 h, followed by immersion in 1% tetrazolium solution (Triphenyl tetrazolium chloride, St. Louis, MO, USA) in the dark at 30°C for 24 h. Subsequently, the seeds were cut in half, with those stained red classified as viable and those unstained classified as dead, and the results were expressed as a percentage.

### 2.3 Seed water absorption

To determine the presence of PY based on the initial moisture content and water absorption, a water absorption test was conducted. The weight of 100 seeds was measured in four replicates and subsequently soaked in distilled water at 4°C in the dark. The surface moisture of the seeds was removed using paper towels every 24 h, and the seeds were weighed. This process was repeated for seven days, with the seeds re-immersed in distilled water after each measurement. Following the final measurement, the seeds were placed in mesh bags and dried in an oven at 70°C for 48 h to determine the initial moisture content based on the dry weight.

### 2.4 Effect of temperatures on embryo growth and germination

To determine the temperatures required for embryo growth, seeds were sown at various temperatures and observed. The seeds were surface-sterilized by soaking them in a 1.4% sodium hypochlorite solution for 10 min and subsequently rinsing them with distilled water. The sterilized seeds were placed on two layers of filter paper in Petri-dishes (10010; SPL Life Science Co. Ltd, Korea), with 4 mL of distilled water, and 50 seeds were sown in six replicates. Embryo growth was observed at constant temperatures of 4, 15, 20, 25, and 30°C [30 µmol·m^-2^·s^-1^ photosynthetic photon flux density (PPFD), white-light emitting diode (LED)] under light conditions, and at alternating temperatures of 15/6 (14/10 h), 20/10 (14/10 h), and 25/15°C (16/8 h) (30 µmol·m^-2^·s^-1^ PPFD, white-LED). The E:S ratio was measured daily at all temperatures by cutting cross sections with a razor blade, and embryo growth was observed for 30 days.

To observe germination characteristics and determine the presence of PD, an initial germination test was conducted. The germination test involved sowing 25 seeds in four replicates at constant temperatures of 4, 15, 20, 25, and 30°C under light or dark conditions and at alternating temperatures of 15/6, 20/10, and 25/15°C. Germination was observed daily for 30 days under light or light/dark cycle conditions, and the final germination (FG, %) was calculated for seeds sown under dark conditions. Additionally, seeds that did not germinate in any of the experiments were tested for viability using 1% tetrazolium solution to distinguish between viable and non-viable seeds.

### 2.5 Expansion of germination temperatures range through gibberellins treatment

To break dormancy and expand the germination temperature range, GAs treatment was conducted. GA_3_ (Cas No. 77-06-5; Sigma-Aldrich, St. Louis, MO, USA) and GA_4 + 7_ (2:1, Cat No. 468-44-0/510-75-8; Kisan Bio, Seoul, Korea) were prepared at concentrations of 0, 100, 200, and 500 mg·L^-1^. The GAs were initially dissolved in a small amount of 100% ethanol and distilled water was subsequently added to achieve the desired concentrations. *L. insularis* seeds were immersed in each GAs solution and treated for 24 h at 4°C in the dark. After GAs treatment, the seeds were sown in four replicates of 25 seeds each at constant temperatures of 4, 15, 20, 25, and 30°C under light conditions and at alternating temperatures of 15/6, 20/10, and 25/15°C under light/dark cycle conditions. Germination was observed daily for 30 days to determine the effects of GAs.

### 2.6 Expansion of germination temperatures range through stratification

Stratification was conducted to determine whether it can expand the germination temperature range of *L. insularis* seeds, whose embryos were grown through stratification. The experimental seeds were placed on two layers of filter paper in Petri-dishes, containing 4 mL of distilled water. Twenty-five seeds were sown in four replicates and subsequently stratifed at 15°C under light conditions for 0, 1, or 2 weeks. Distilled water was added regularly to prevent drying during the stratification period. After stratification, the seeds were transferred to constant temperatures of 4, 15, 20, 25, and 30°C under light conditions, and at alternating temperatures of 15/6, 20/10, and 25/15°C under light/dark cycle conditions. Germination was observed daily for 30 days, and the FG (%), mean germination time (MGT, days), and time for 50% germination (T_50_, days) were calculated. The germination indices were calculated using the following formula:


$$Final\;germination\;\left(\%\right)=\left(\frac{Germinated\;seeds}{Total\;seeds}\right)\times 100$$

$$Mean\;germination\;time\;\left(days\right)=\frac{\sum \left(n_{i}\times t_{i}\right)}{\sum n_{i}}$$

Where $n_{i}$ is the number of seeds germinated at time $t_{i}$ .


$$T_{50}\;\left(days\right)=t_{b50}+\frac{\frac{N}{2}-G_{b50}}{G_{a50}-G_{b50}}\times \left(t_{a50}-t_{b50}\right)$$

Where $t_{b50}$ is the time just before 50% germination, N is the total number of germinated seeds, $G_{a50}$ is the cumulative number of seeds germinated just after 50%, $G_{b50}$ is the cumulative number of seeds germinated just before 50%, and $t_{a50}$ is the time just after 50% germination.

### 2.7 Changes in germination temperatures range through dormancy-release (DR) priming

DR priming was conducted to apply priming to dormant seeds and confirm the commercial potential of seeds with expanded germination temperature range. DR priming involved stratifying *L. insularis* seeds at 15°C under light conditions for 1 and 2 weeks, followed by drying at room temperature for 48 h to restore their initial moisture content. Subsequently, untreated seeds (control), seeds stratified at 15°C under light conditions for 1 week (1W) or 2 weeks (2W), and seeds that completed DR priming for 1 week (1DR) or 2 weeks (2DR) were sown under light/dark cycle conditions at 20/10 and 25/15°C. Germination was observed daily for 30 days, and the FG, MGT, and T_50_ were calculated.

To confirm seedling emergence in the soil, 25 seeds each from the control, 1DR, and 2DR priming seeds groups were sown in four replicates in transparent plastic boxes filled with horticultural soil (Hs, Hanareum No.2; Shinsung Mineral Co., Ltd., Seongnam, Korea). The seeds were sown under light/dark cycle conditions at 20/10 (14/10 h) and 25/15°C (16/8 h). Seedling emergence was calculated at 4 and 8 weeks.

### 2.8 Statistical analyses

The weight of 100 seeds, size of seeds, E:S ratio, and germination experiments (FG, MGT, and T_50_), and seedling emergence are expressed as the mean and standard error (SE). Significance was assessed using analysis of variance to test the null hypothesis, followed by Duncan’s Multiple Range Test to compare the means, with a significance level of *p*< 0.05. Analyses were performed using the Statistical Analysis Software (SAS, Version 9.4; SAS Institute Inc., Cary, NC, USA).

## 3 Results

### 3.1 Seed basic information

**Figure 1:**
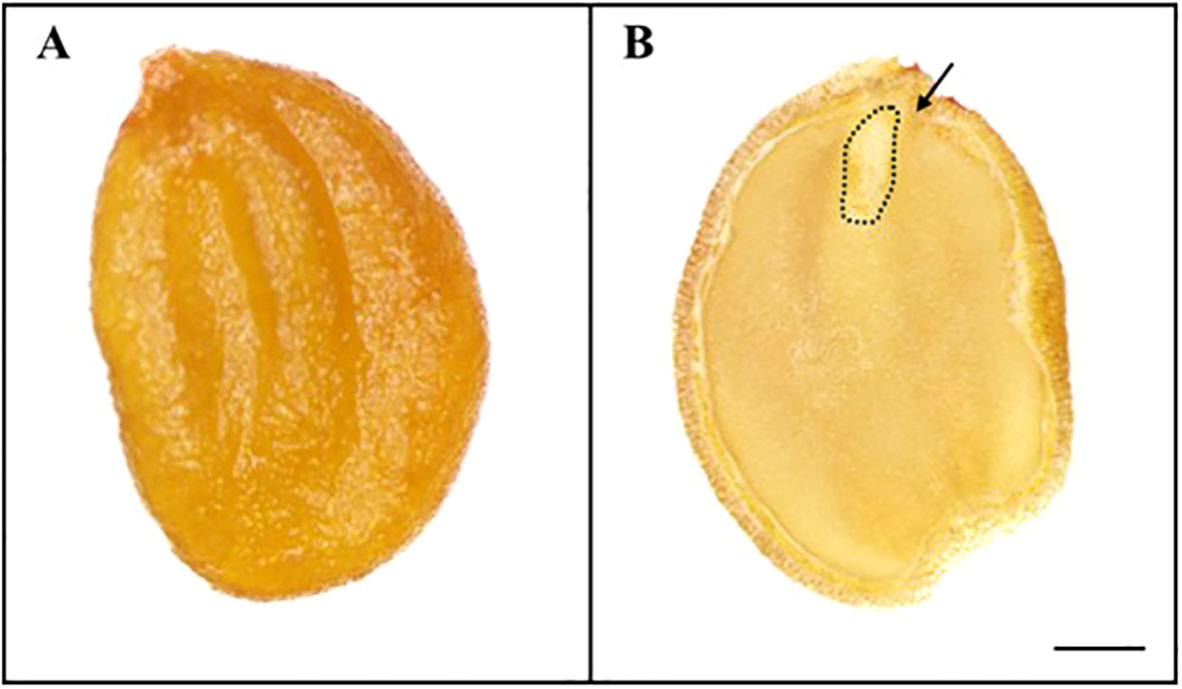
Stereomicroscope images of *Lonicera insularis* seed. **(A)**, appearance of the seed; **(B)**, cross-sections of seed. Scale bar = 1 mm. Arrows indicate embryo.

**Figure 2:**
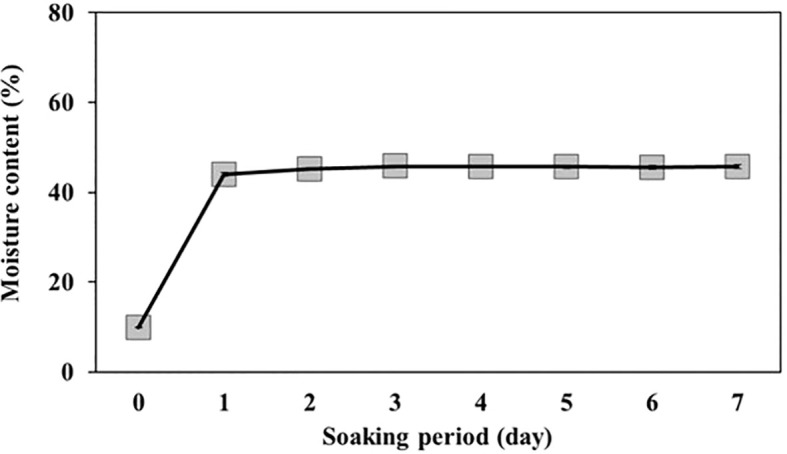
Changes in moisture content of *Lonicera insularis* seeds during the soaking period. Bars represent standard errors (n = 4).

The weight of 100 *L. insularis* seeds measured 0.44 ± 0.01 g, with a seed length of 3.87 ± 0.07 mm and a seed width of 2.64 ± 0.05 mm (**Table 1**, **Figure 1**). Seeds exhibited an E:S ratio of 0.24 ± 0.01, indicating an underdeveloped embryo, whereas the E:S ratio of seeds immediately before germination was 0.81 ± 0.05. A significant difference was observed between the seeds and seeds immediately before germination. The seed viability test using a 1% tetrazolium solution indicated 84.0% viability. The initial moisture content of *L. insularis* seeds was 9.83 ± 0.07%, reaching a maximum moisture content of 43.9% on the first day of water absorption (**Figure 2**).

**Table 1:** Basic information of the collected *Lonicera insularis* seeds used in this study.

Weight^z^	Size^y^		E:S ratio^x^	Viability (%)
	Length (mm)	Width (mm)		
0.44 ± 0.01	3.87 ± 0.07	2.64 ± 0.05	0.24 ± 0.01	84.0

^z^Weight of 100 seeds, ^y^Size (length and width) of seeds, ^c^Embryo (E) length: Seed (S) length ratio. Seed weight, size and E:S ratio data represent the mean ± standard error.

### 3.2 Effect of temperatures on embryo growth and germination

**Figure 3:**
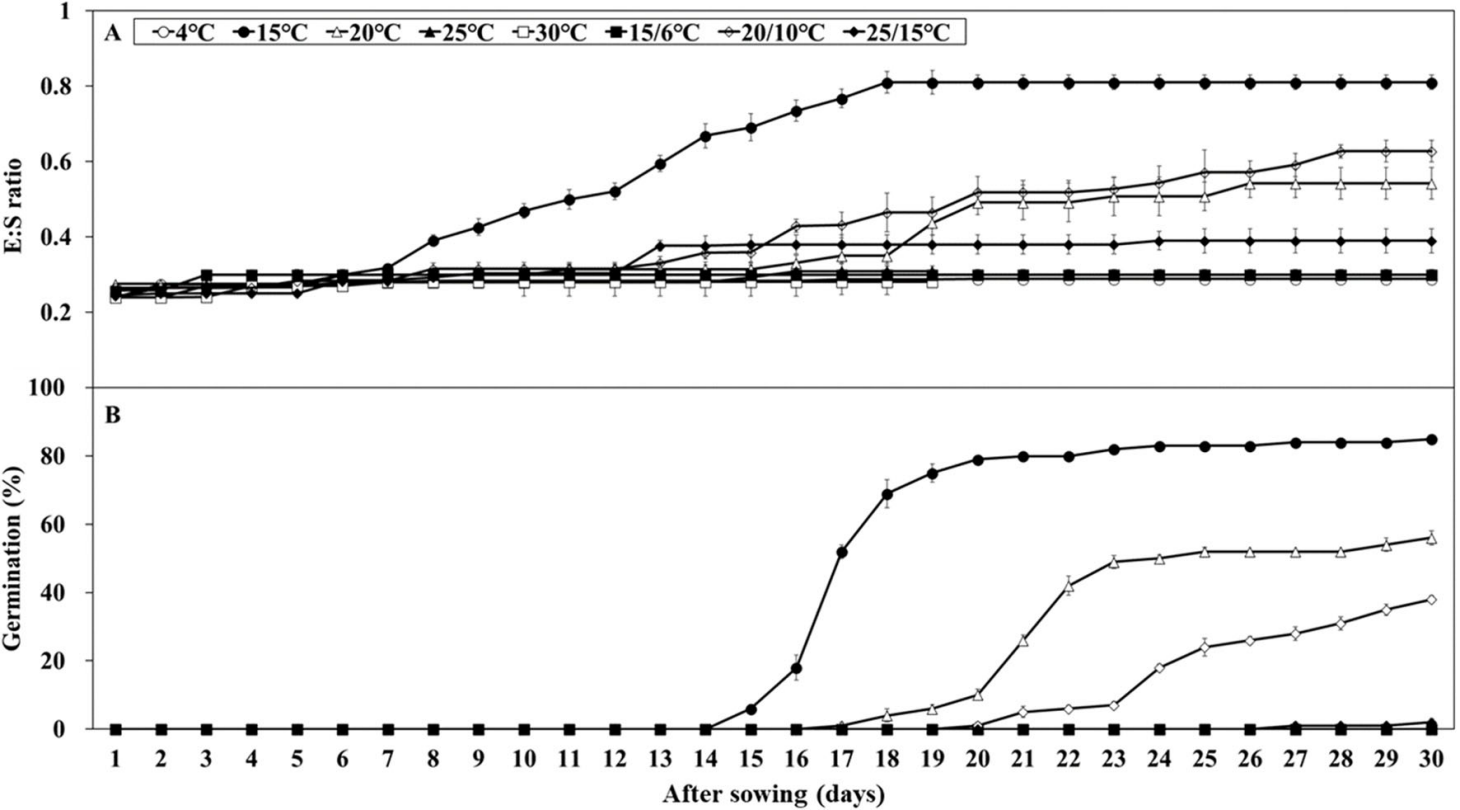
Embryo growth and percent germination of *Lonicera insularis* seeds at various temperatures under light and light/dark conditions. **(A)**, E:S ratio; **(B)**, germination. Bars represent standard errors.

Embryo growth was monitored over 30 days at various temperatures (15, 20, 20/10, and 25/15°C), whereas no growth was observed at other temperatures (**Figure 3A**). After 30 days, the E:S ratio ranged from 0.39 – 0.62 at 20, 20/10, and 25/15°C. At 15°C, the E:S ratio reached 0.81 after 18 days, indicating complete embryo growth. Initial germination test results indicated germination ranging from 0.0 – 85.0% under light and light/dark cycle conditions, whereas no germination was observed under dark conditions (**Figure 3B**). At 20, 20/10, and 25/15°C, germination was 52.0, 38.0, and 2.0%, respectively, which did not reach the viability level (84.0%). The FG at 15°C was 85.0%, which is significantly higher than that at all other temperatures. Therefore, 15°C under light conditions was identified as optimal for embryo growth and germination and was used in subsequent stratification experiments.

### 3.3 Effect of gibberellins treatment on seed dormancy

**Figure 4:**
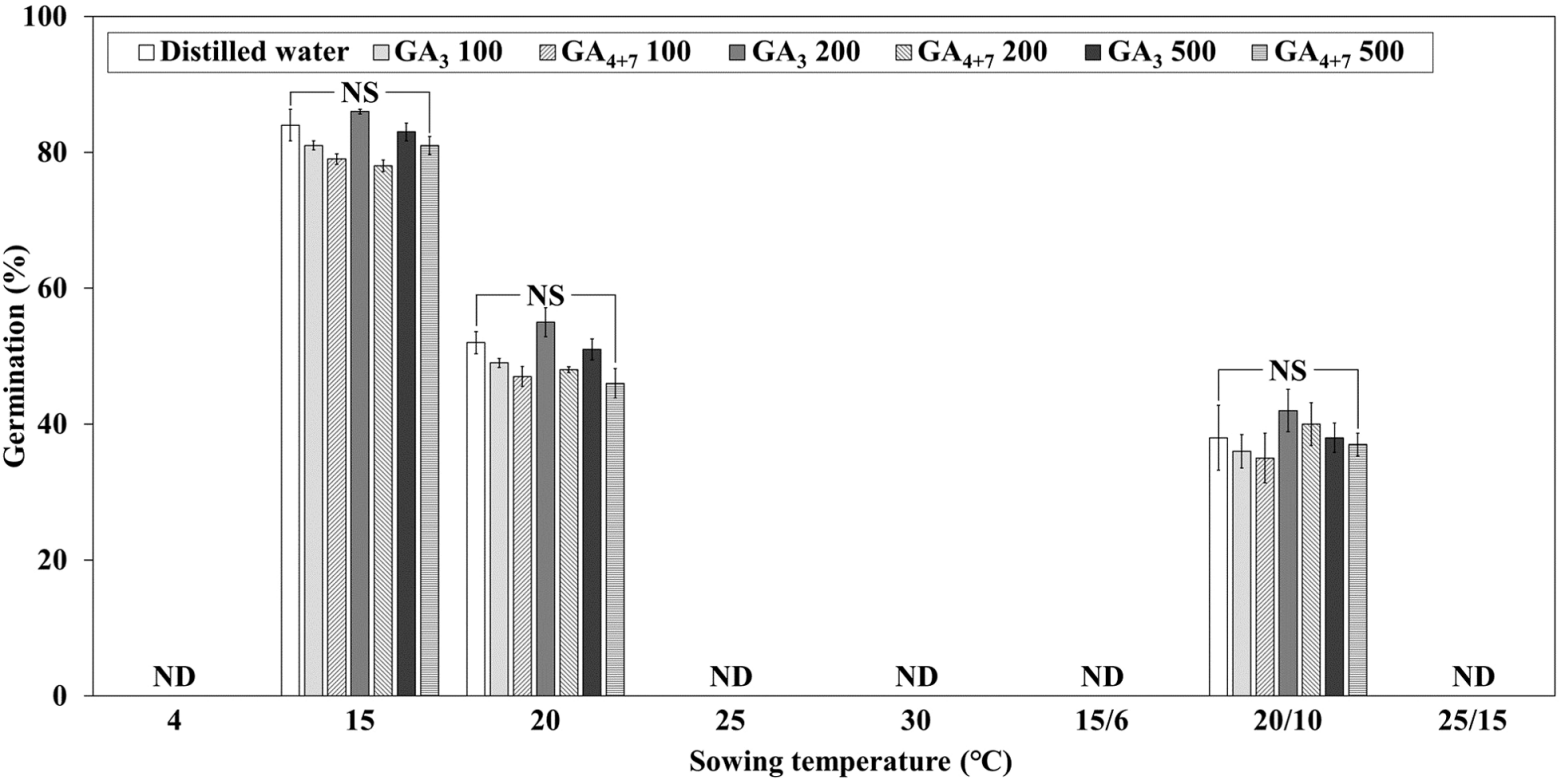
Final germination percentages of *Lonicera insularis* seeds treated with different GAs concentration for 30 days at various temperatures under light and light/dark conditions. ND, not detected seedling emergence; NS, no significant differences in concentrations of GA_3_ and GA_4 + 7_ at each temperature as determined by Duncan’s multiple range test (*p<* 0.05). Bars represent standard errors (n = 4).

Treatment of *L. insularis* seeds with 0, 100, 200, and 500 mg·L^-1^ of GA_3_ and GA_4 + 7_ for 24 h did not significantly affect dormancy breaking and germination temperature range (**Figure 4**). Germination was observed only at 15, 20, and 20/10°C, with FG ranging from 46.0 – 55.0% at 20°C and 35.0 – 42.0% at 20/10°C; however, no germination was observed at other temperatures. In contrast, FG was the highest at 15°C, ranging from 78.0 – 86.0%, but no significant differences were observed among the concentrations at any temperature.

### 3.4 Effect of stratification periods on germination temperature range

**Figure 5:**
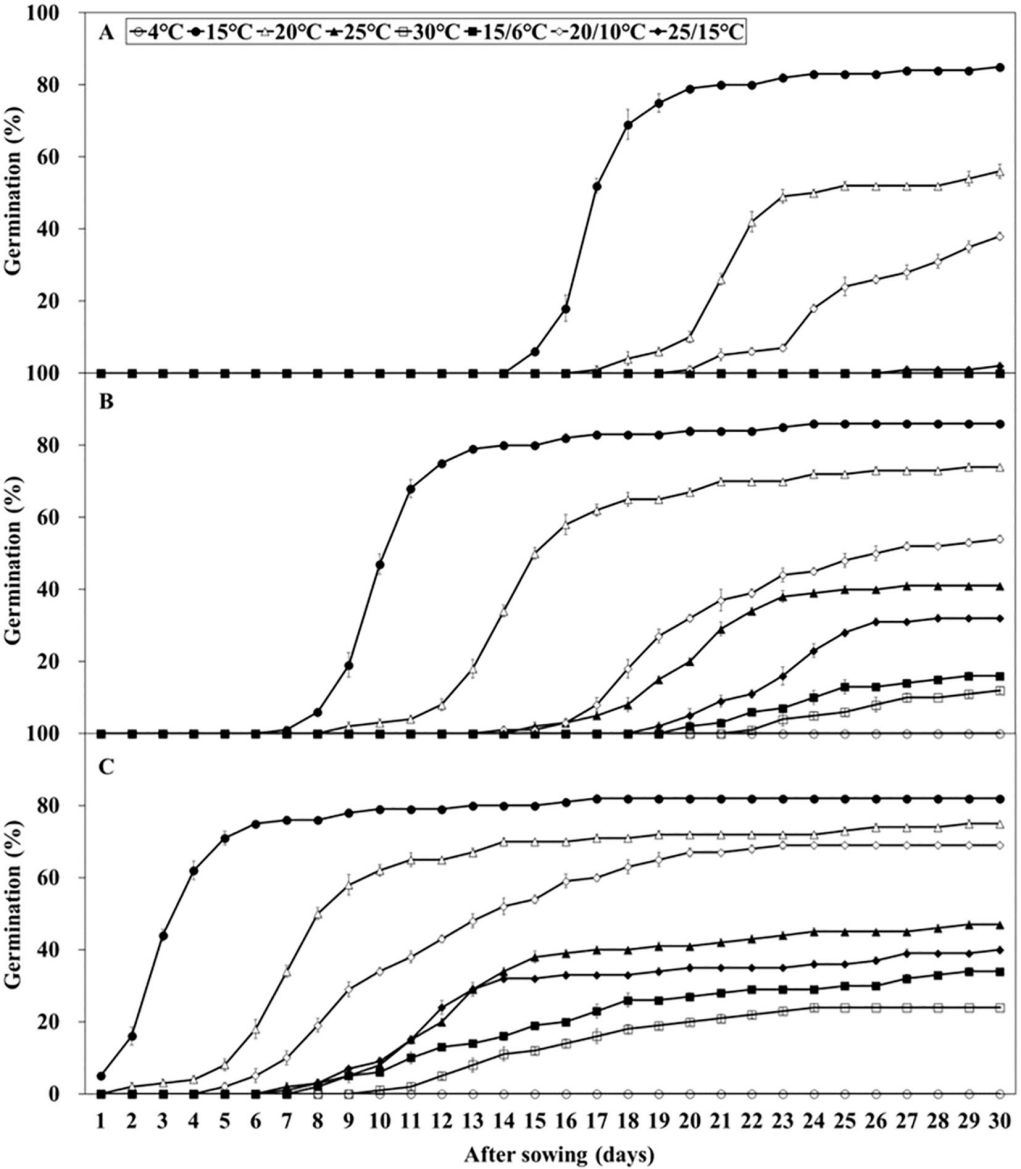
Percent germination of *Lonicera insularis* seeds at various temperatures after stratification 0, 1, and 2 weeks at 15°C under light condition. **(A)**, stratification 0 week at 15°C; **(B)**, stratification 1 week at 15°C; **(C)**, stratification 2 weeks at 15°C. Bars represent standard errors (n = 4).

**Figure 6:**
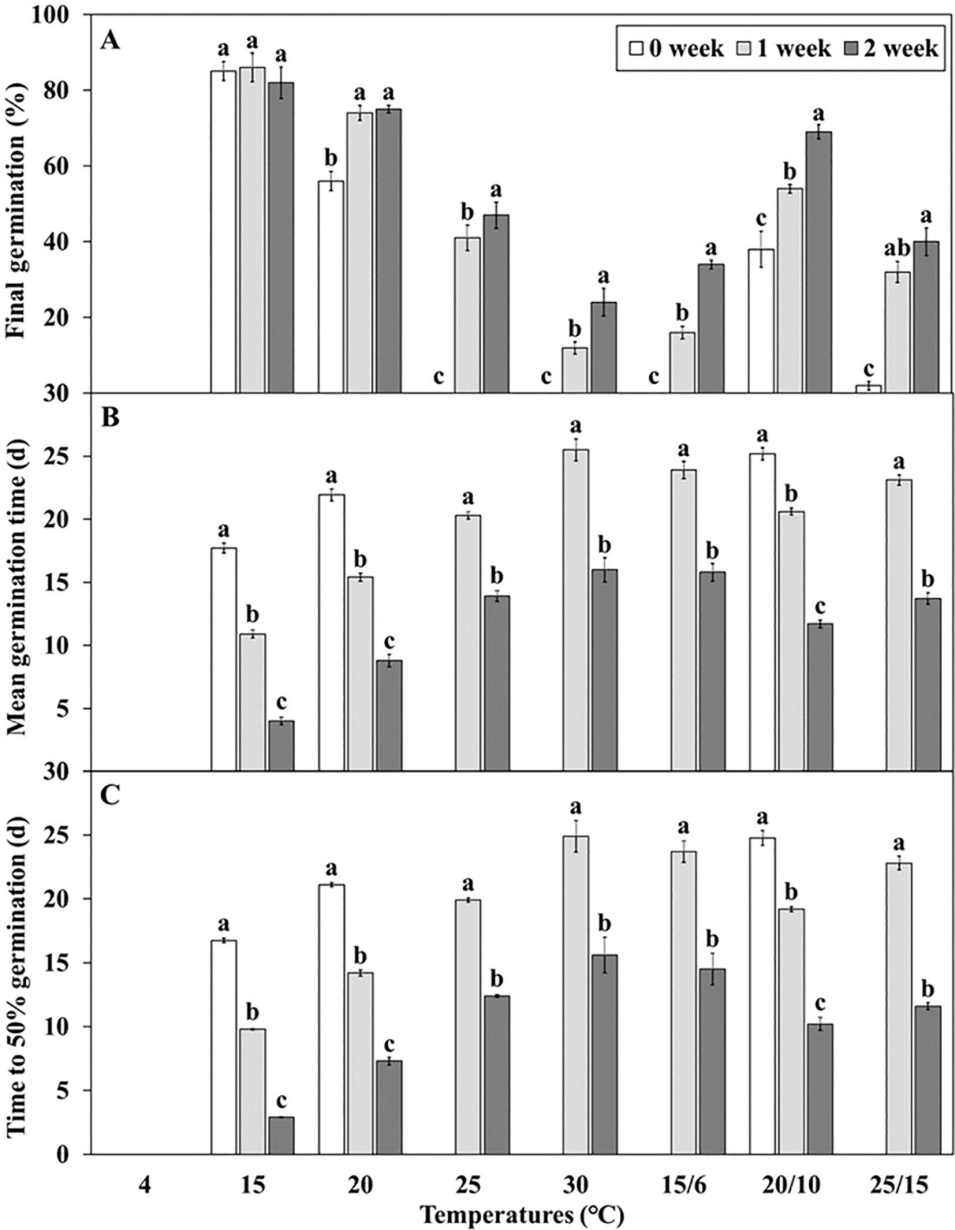
Germination characteristics of *Lonicera insularis* seeds for 30 days at various temperatures after stratification 0, 1, and 2 weeks at 15°C under light condition. **(A)**, final germination; **(B)**, mean germination time; **(C)**, time to 50% germination. Different letters indicate significant differences in each temperature across stratification periods as determined by Duncan’s multiple range test (*p*< 0.05). Bars represent standard errors (n = 4).

*L. insularis* seeds began embryo growth at 7 days at 15°C, completing the growth by 18 days with an E:S ratio of 0.81; however, germination started at 15 days (**Figure 3**). Therefore, stratification was conducted for 14 days, from the onset of embryo growth (7 days) until immediately before germination. Stratification of *L. insularis* seeds at 15°C for 0, 1, and 2 weeks resulted in different patterns across various temperatures, based on the stratification period (**Figures 5**, **6**). A longer stratification period at 15°C significantly increased FG and reduced MGT and T_50_. Seeds with 0 week of stratification at 15°C exhibited germination ranging from 2.0 – 85.0% at certain temperatures, including 15, 20, 20/10, and 25/15°C (**Figures 5A**, **6A**). In contrast, seeds stratified for 1 week at 15°C exhibited germination ranging from 12.0 – 86.0% at all temperatures, except at 4°C (**Figures 5B**, **6A**). Stratification for 2 weeks at 15°C significantly expanded the germination temperature range, with germination ranging from 24.0 – 82.0% at all temperatures, except at 4°C and significantly increased the FG at 20, 25, 30, 15/6, 20/10, and 25/15°C (**Figures 5C**, **6A**). Additionally, seeds stratified for 2 weeks at 15°C significantly facilitated the germination rates regarding MGT and T_50_ (**Figures 6B, C**).

### 3.5 Effect of dormancy-release priming on germination and seedling emergence

**Figure 7:**
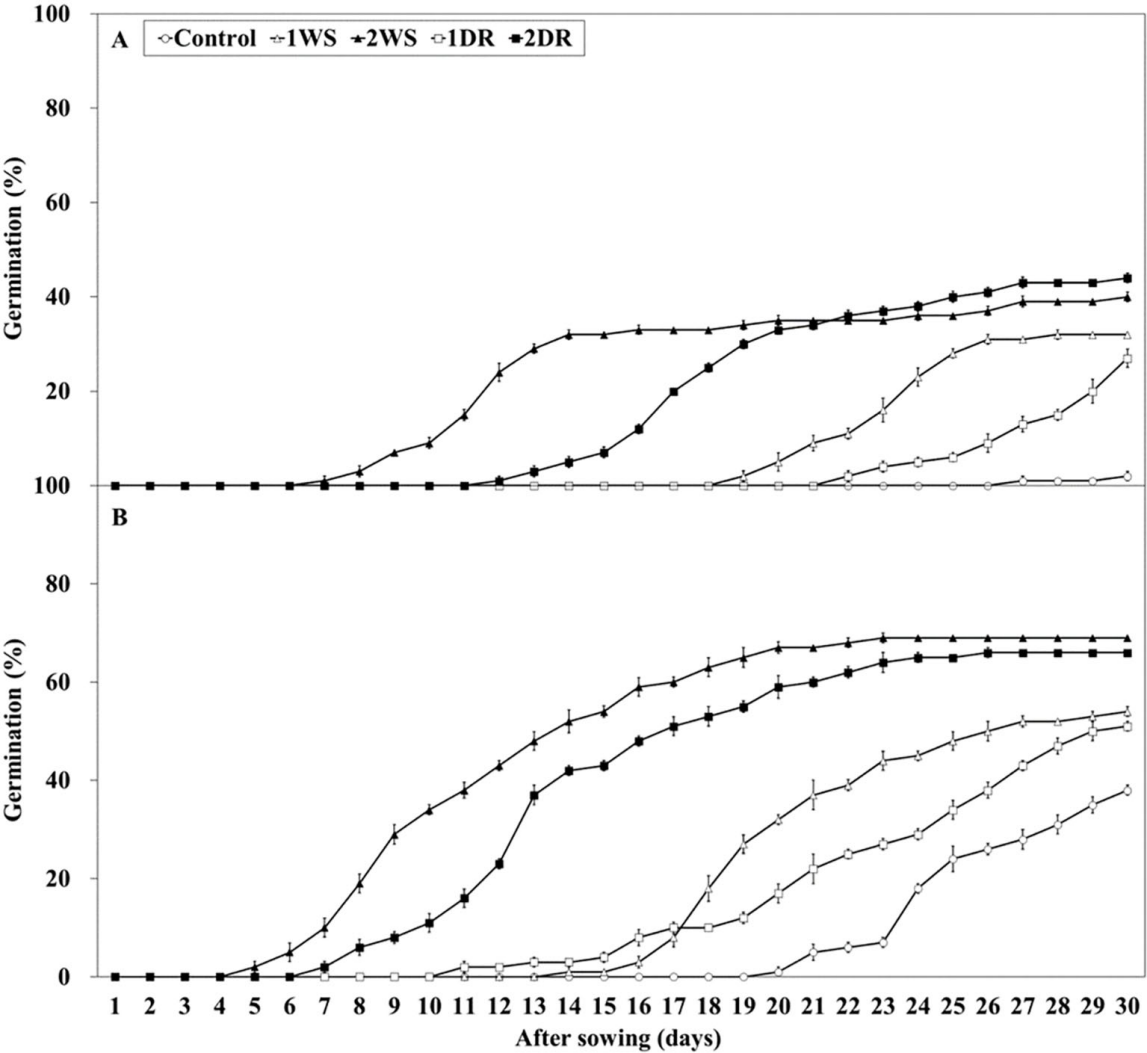
Percent germination of *Lonicera insularis* seeds for 30 days at 25/15 and 20/10°C after stratification and dormancy-release priming. **(A)**, 25/15°C; **(B)**, 20/10°C. Control, non-treatment; 1WS, stratification at 15°C for 1 week; 2WS, stratification at 15°C for 2 weeks; 1DR, stratification at 15°C for 1 week and drying for 2 days; 2DR, stratification at 15°C for 2 weeks and drying for 2 days. Bars represent standard errors (n = 4).

**Figure 8:**
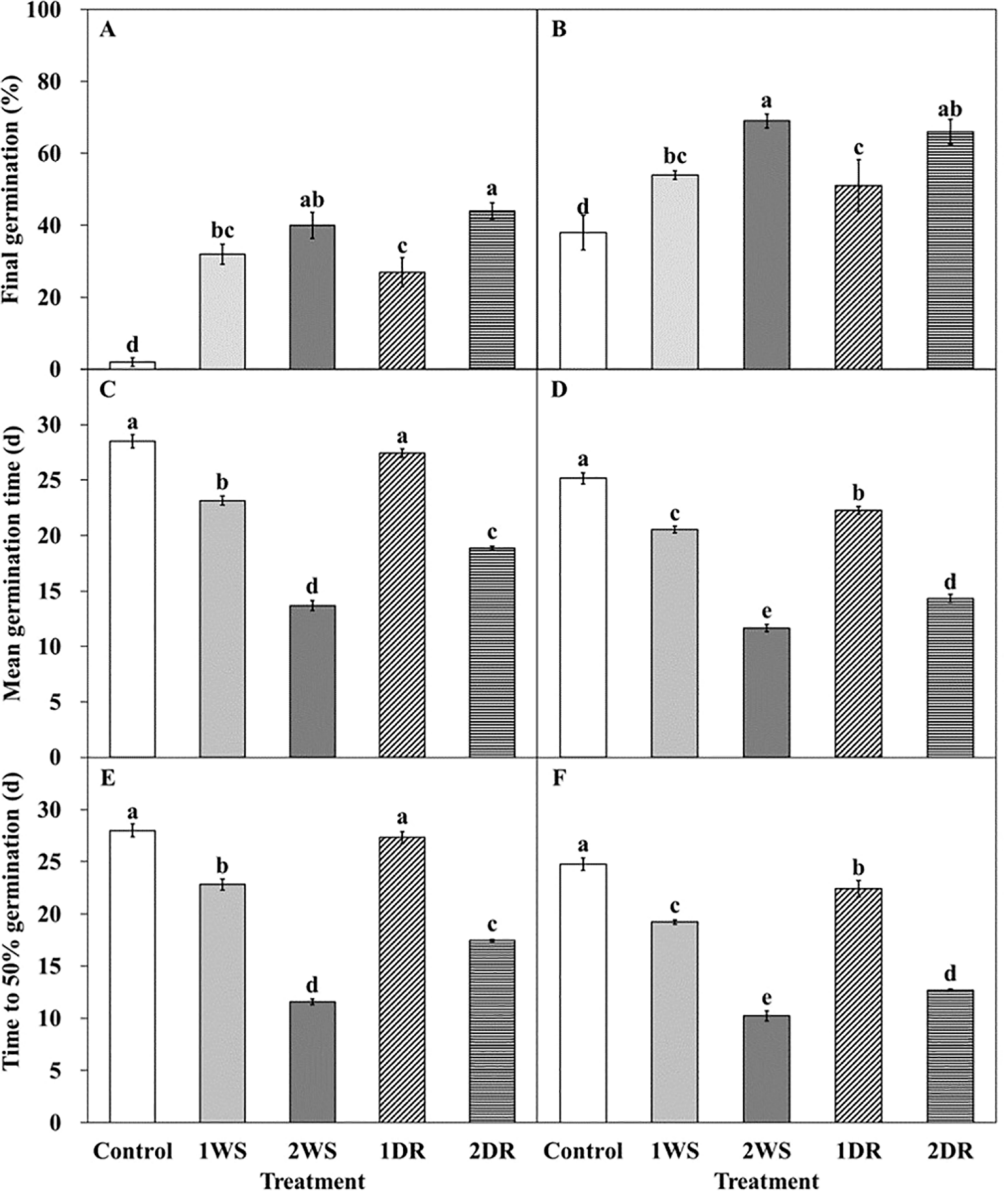
Germination characteristics of *Lonicera insularis* seeds for 30 days under 25/15 and 20/10°C according to stratification and dormancy-release priming. **(A, C, E)**, 25/15°C; **(B, D, F)**, 20/10°C. Control, non-treatment; 1WS, stratification at 15°C for 1 week; 2WS, stratification at 15°C for 2 weeks; 1DR, stratification at 15°C for 1 week and drying for 2 days; 2DR, stratification at 15°C for 2 weeks and drying for 2 days. Different letters indicate significant differences in each temperature across treatments as determined by Duncan’s multiple range test (*p<* 0.05). Bars represent standard errors (n = 4).

Significant differences in FG, MGT, and T_50_ were observed among the control, 1WS, 2WS, 1DR, and 2DR treatments at 20/10 and 25/15°C (**Figures 7**, **8**). FG ranged from 2.0 – 44.0 and 38.0 – 69.0% at 25/15°C, and 20/10°C. At both 20/10 and 25/15°C, all treatments exhibited significantly higher FG than that of the control. Additionally, longer stratification periods enhanced FG, MGT, and T_50_ in all the treatments (**Figure 8**). FG for 1WS and 1DR at 20/10°C was 54.0 and 51.0%, respectively, with no significant difference in FG. In contrast, the MGT and T_50_ values for 1WS were 20.6 and 19.2, respectively, whereas those for 1DR were 22.3 and 22.4, respectively, indicating delayed germination rates for 1DR. At 25/15°C, the FG values for 1WS and 1DR were 32.0 and 27.0, respectively, indicating no significant difference. However, the MGT for 1WS and 1DR was 23.15 and 27.4, respectively, and the T_50_ was 22.8 and 27.3, respectively, indicating a significantly delayed germination rate for 1DR. Similarly, no significant difference was observed in FG between 2WS and 2DR at 20/10 and 25/15°C, but 2DR exhibited delayed germination rates regarding MGT and T_50_.

**Figure 9:**
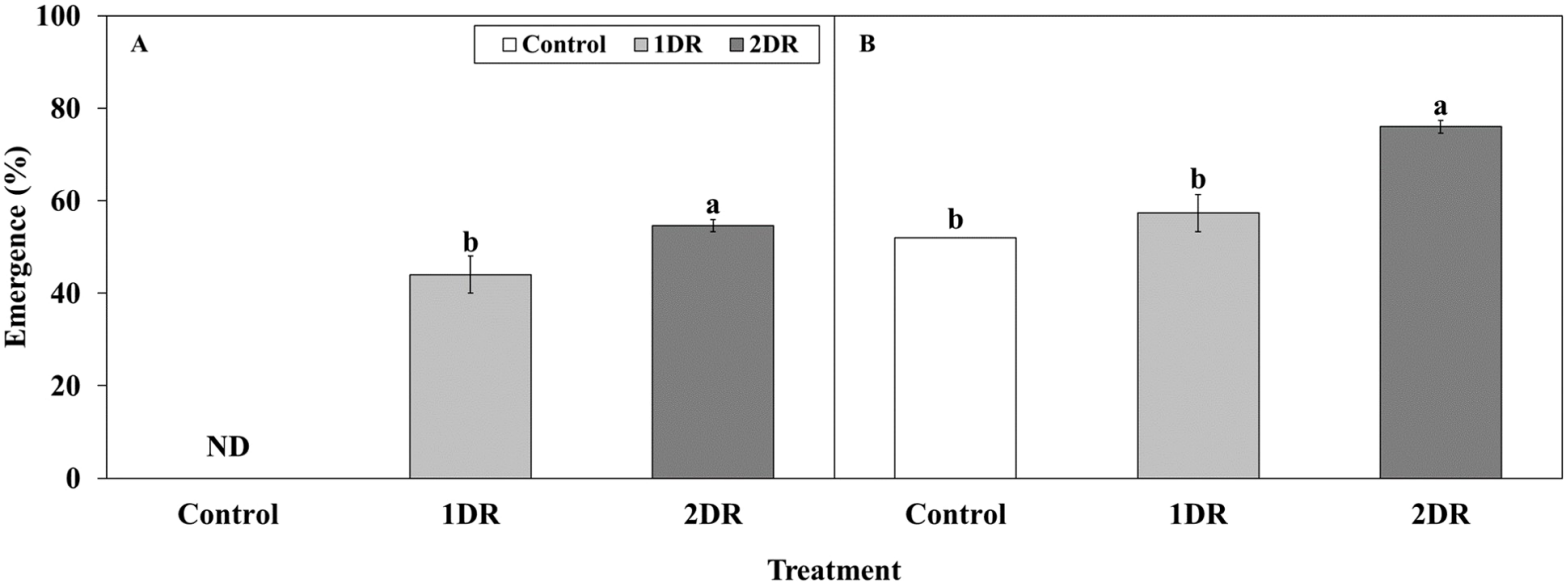
Seedling emergence of *Lonicera insularis* seeds DR-primed for different periods. **(A)**, seedling emergence at 4 weeks; **(B)**, seedling emergence at 8 weeks. Control, non-treatment; 1DR, stratification at 15°C for 1 week and drying for 2 days; 2DR, stratification at 15°C for 2 weeks and drying for 2 days; ND, not detected seedling emergence. Different letters indicate significant differences among treatments as determined by Duncan’s multiple range test (p < 0.05). Bars represent standard errors (n = 4).

At 20/10°C, seedling emergence was observed in the control, 1DR, and 2DR treatments. At 4 weeks, seedling emergence in the control, 1DR, and 2DR was 0, 44.0, and 54.7%, respectively, with significantly higher emergence observed in 2DR (**Figure 9A**). By 8 weeks, the emergence in the control, 1DR, and 2DR was 52.0, 57.3, and 76.0%, respectively, with significantly higher emergence in 2DR (**Figure 9B**).

## 4 Discussion

### 4.1 *L. insularis* seeds have morphological dormancy

An underdeveloped embryo is characterized by a relatively small embryo size and an E:S ratio of< 0.5, and is classified as MD (Baskin and Baskin, 2014). Seeds with MD have embryos that are either undifferentiated or underdeveloped at harvest, necessitating time for further development before germination (Rhie et al., 2016). The seeds of *L. insularis* exhibited an E:S ratio of 0.24, indicating an underdeveloped embryo, thereby confirming the presence of MD. These underdeveloped embryos of *L. insularis* seeds were able to achieve an E:S ratio of 0.81 through incubation at 15°C under light conditions. Seed water absorption is the result of long-term adaptation to natural habitats and represents the initial stage of seed germination (Yang et al., 2023). Seeds with PY, such as *Vicia villosa* (Renzi et al., 2016), *Senna multijuga* (Rodrigues-Junior et al., 2018), *Delonix regia* (Jaganathan et al., 2017), and *Melilotus albus* (Zhang et al., 2020), often encounter hindrance in water uptake owing to their impermeable seed coat that negatively affects seed germination. PY is caused by the impermeability of palisade cells in the seed or fruit coats (Tang et al., 2022). Seeds are considered permeable if their weight increases by over 20% upon water absorption (Baskin and Baskin, 2004). Because *L. insularis* seeds reached a maximum moisture content of 49.9% on the first day of water absorption, the characteristics of PY owing to impermeability were not observed.

Seeds with PD do not germinate within 4 weeks, even after water absorption (Baskin and Baskin, 2022). Approximately 70% of the seed plants produce dormant seeds, and PD is the most common class of dormancy (Soltani et al., 2017). However, *L. insularis* seeds exhibited 85.0% germination within 30 days at 15°C, indicating the absence of PD characteristics. In general, seeds with only MD complete embryo growth in a relatively short period and germinate within 4 weeks (Baskin et al., 2008). Additionally, seeds with MD require appropriate temperatures for embryo growth and germination (Soltani et al., 2019). According to previous studies, *L. insularis* seeds have underdeveloped embryos and were classified as MD, with 94.4% germination at 15°C in 4 weeks (Park et al., 2019). In this study, the *L. insularis* seeds also demonstrated embryo growth at 15°C, with germination observed from day 15, indicating the absence of PD. Therefore, the seeds of *L. insularis* were classified as exhibiting only MD, with underdeveloped embryos and germination within 4 weeks at 15°C.

In this study, *L. insularis* seeds germinated only under light conditions. Although the seeds of numerous species successfully germinate in both light and darkness, certain species may germinate only under light or dark conditions (Baskin and Baskin, 2014). Light serves as a crucial environmental signal that controls diverse biological processes, such as seed dormancy and germination, photomorphogenesis, phototropism, shade avoidance, and flowering (Yang et al., 2020). Additionally, light is an important environmental factor that helps seeds determine whether the surrounding environment is suitable for germination and subsequent seedling emergence (Yan and Chen, 2020). Therefore, the role of light in regulating the germination of *L. insularis* seeds highlights the species’ potential reliance on light as a cue for optimal germination conditions.

### 4.2 Stratification can expand germination temperature range of *L. insularis* seeds

Dormant seeds germinate within a narrower temperature range than seeds with alleviated dormancy (Baskin and Baskin, 2014). Additionally, dormant seeds germinate only within an appropriate temperature range (Geneve, 2003). Therefore, further research is required to expand the germination temperature range of dormant seeds for their utilization. The germination temperature range of *Ribes multiflorum* seeds was expanded through GA_3_ treatment (Mattana et al., 2012). However, GA treatment did not significantly affect the germination of *L. insularis* seeds. This may be because of the presence of high levels of abscisic acid (ABA), which maintains dormancy (Finch-Savage and Leubner-Metzger, 2006). Cold or warm stratification breaks dormancy and induces germination through the interaction between various hormones, including ABA and GA (Leon et al., 2007). *L. insularis* seeds germinated only at specific temperatures of 15, 20, 20/10, and 25/10°C. The germination temperature range was enhanced for *L. insularis* seeds that had undergone embryo growth through stratification at 15°C for 0, 1, and 2 weeks because germination was observed at all temperatures, except at 4°C. Additionally, once embryo growth was completed through 2 weeks of stratification at 15°C, germination was effectively enhanced at previously non-germinating temperatures. This indicated that stratification at 15°C alleviated dormancy through the interaction of various hormones, thereby expanding the germination temperature range. In *Pinus koraiensis* seeds, cold stratification effectively expanded the germination temperature range (Song et al., 2023). Moreover, determining the optimal temperature and duration for stratification may help identify methods that can alleviate dormancy in historically challenging species, which can be crucial for restoration efforts (Just et al., 2023). Germination temperature range significantly affects the ecological environment of a species, and species that can germinate over a broader temperature range may have wider geographical distributions (Donohue et al., 2010). Research aimed at expanding the germination temperature range offers an alternative approach to nursery cultivation, thereby addressing climate crises for species conservation and propagation (Song et al., 2023). Therefore, in this study, *L. insularis* seeds effectively expanded their germination temperature range through 2 weeks of stratification at 15°C, suggesting this as a dormancy-breaking method for propagation, conservation, and utilization of the species.

### 4.3 Effect of dormancy-release priming in *L. insularis* seeds

Priming promotes seed germination metabolism, enhances germination, resistance to environmental stress, and dormancy breaking, and enhances seedling production capacity (Malek et al., 2022). Additionally, priming is an effective technique that breaks seed dormancy and enables immediate sowing (Bourioug et al., 2020). Moreover, primed seeds exhibit synchronous and fapid emergence, reduced dormancy, a broader wider range of germination temperatures, and enhanced competitiveness against weeds and pathogens (Paparella et al., 2015). However, despite various studies on enhancing priming, novel ideas are still lacking. Seeds with MD have underdeveloped embryos and require time for the embryos to grow to a critical size before germination (Soltani et al., 2018). Therefore, to apply priming to seeds with MD, the embryo should be grown and subsequently re-dried to complete the priming process. We confirmed that embryo growth occurred at 15°C, and 2 weeks stratification effectively expanded the germination temperature range, which was subsequently used for DR priming. The 2WS and 2DR treatments on *L. insularis* did not demonstrate significant differences in FG at 20/10 and 25/15°C. This indicates that the DR-priming technique, which expands the germination temperature range, may be commercially useful for *L. insularis* seeds.

In this study. the 2DR treatment applied to *L. insularis* seeds with MD demonstrated a slight delay in the germination rate compared to 2WS. For successful seed priming, water absorption should be halted before radicle protrusion; however, re-dried seeds can lose vigor (Pagano et al., 2023). Damage from priming has also been observed in *Digitalis purpurea* seeds, where redrying caused cellular damage, seed aging, and reduced vigor (Butler et al., 2009; Waterworth et al., 2024). Priming activates primary cellular processes before germination, such as the *de novo* synthesis of nucleic acids and proteins, ATP production, and DNA repair (Paparella et al., 2015). Seed repair response plays a crucial role in maintaining vigor, and DNA repair functions should be maintained at appropriate levels in the embryo to sustain seed vitality (Rajjou and Debeaujon 2008; Oge et al., 2008; Paparella et al., 2015). When DNA damage is effectively repaired, embryo cells resume the cell cycle progression and replicate DNA, but defective repair mechanisms, result in oxidative injury that causes cell death (Waterworth et al., 2010; Kranner et al., 2010; Balestrazzi et al., 2011; Ventura et al., 2012). Therefore, the delayed germination rate of DR-primed *L. insularis* seeds may be because of damage from redrying, and further research is required to address this issue, including identifying the specific causes of delayed germination resulting frome the priming process. Seed invigoration techniques, such as priming, shortens the seedling emergence time and achieves uniform seedling emergence, offering beneficial effects for numerous horticultural and agricultural crops (Jisha et al., 2013). In this study, 2DR-treated *L. insularis* seeds exhibited significantly higher seedling emergence rates at 4 and 8 weeks at 20/10°C. This indicates that DR-primed seeds with an expanded germination temperature range can be commercially useful. Therefore, research on seed propagation to secure seeds with dormancy for ornamental and medicinal purposes should continue, and the novel approach of DR priming technology can be applied to resource plant seeds with dormancy similar to *L. insularis* seeds.

## 5 Conclusions

In conclusion, *Lonicera insularis* seeds germinate within 4 weeks but have an underdeveloped embryo and are therefore classified as MD. The embryos of *L. insularis* seeds completed growth through stratification at 15°C, and 2 weeks of stratification at 15°C effectively expanded the germination temperature range. Additionally, the DR priming technology applied to *L. insularis* seeds using stratification to break dormancy expanded the germination temperature range and can be commercially useful, although further research is required to enhance the germination rate. This priming technology can be applied to various species with MD, and generating additional knowledge of novel seed priming methods, such as DR priming, can significantly contribute to the agricultural industry. Additionally, research should focus on applying DR priming to species with other dormancy types (e.g., PD, PY, MPD, and PY+PD) to support seed propagation, restoration, and utilization.

## Data Availability

The datasets presented in this study can be found in online repositories. The names of the repository/repositories and accession number(s) can be found in the article/supplementary material.
